# Supplementary material for: Systems approach reveals photosensitivity and PER2 level as determinants of clock‐modulator efficacy
Source: Mol Syst Biol. 2019 Jul 8;15(7):e8838. doi: 10.15252/msb.20198838 (PMC6613017; doi:10.15252/msb.20198838)
Supplement: Supplementary file 1 — Appendix [file MSB-15-e8838-s001.docx]

**Appendix:** **Systems approach reveals photosensitivity and PER2 level as determinants of clock-modulator efficacy**

Dae Wook Kim^1^, Cheng Chang^2*^, Xian Chen^3^, Angela Doran^4^, Francois Gaudreault^5^, Travis Wager^6^, George J. DeMarco^7^, Jae Kyoung Kim^1*^

1. Department of Mathematical Sciences, Korea Advanced Institute of Science and Technology, Daejeon, Republic of Korea.

2. Systems Modeling and Simulation Group, Pharmacokinetics, Dynamics and Metabolism, Worldwide Research & Development, Pfizer Inc, Groton, Connecticut, USA.

3. Comparative Medicine, Worldwide Research & Development, Pfizer Inc, Cambridge, Massachusetts, USA.

4. Enzymology and Transporter Group, Pharmacokinetics, Dynamics and Metabolism, Worldwide Research & Development, Pfizer Inc, Groton, Connecticut, USA.

5. Clinical Pharmacology and Pharmacometrics, Research & Development, Biogen Inc, Cambridge, Massachusetts, USA.

6. Neuroscience Research Unit, Worldwide Research & Development, Pfizer Inc, Boston, Massachusetts, USA.

7. Department of Animal Medicine, University of Massachusetts Medical School, Worcester, Massachusetts, USA.

**Table of contents**

**Appendix Equation S1. The ordinary differential equations of the mathematical model.**

**Appendix Equation S1. The ordinary differential equations of the mathematical model (Fig 2A).** The newly modified parameters (Fig EV1A-C and Dataset EV1) and the gating and adaptation for light (Fig 2Aii and EV1F-H) are highlighted in red. Note that as the function g and p (Fig EV1F and G) were constructed using *Interpolation*, which is a built-in function in *MATHEMATICA*, they cannot be explicitly written (see Materials and Methods). Thus, for details of the construction of g and p, see code EV1. Furthermore, the variables and parameters are described in Table EV1 and 2 and Dataset EV1 and 2. The equations of the original model are adopted from the supplementary information in (Kim et al, 2013).

**Kinetics and dynamics of PF-670462**

pInh'==-(uinp+nlpin)*pInh+nepin*bInh bInh'=(nlbin+nepin)*bInh+nlpin*pInh+nebin*Inh

Inh'=nlbin*bInh-(nebin+nlin)*Inh+nein*nInh+(-inbin*Inh

*Sum[x[jj][kk][ll][0][0],{jj,0,6},{kk,0,2},{ll,1,3,2}])+inubin*Sum[x[jj][kk][ll][0][0],{jj,0,6},{kk,0,2},{ll,4,5}]

nInh'== Inh*nlin-(nein)* nInh-

(inbin*Nf*nInh* Sum[x[jj][kk][ll][1][nn],{jj,0,6},{kk,0,2},{ll,1,3,2},{nn, 0,1}])

+inubin*Sum[ x[jj][kk][ll][1][ nn],{jj,0,6},{kk,0,2},{ll,4,5},{nn,0,1}]

**Light Activity**

**When light is on (T=Light duration (h))**

MnPo'=trPo*G-tmc*MnPo- umPo*MnPo+trPo*lono*g(p(revng,revnp))*(Z1^Z2^/(T^Z2^+Z1^Z2^))

MnPt’=trPt*G-tmc*MnPt-umPt*MnPt+trPt*lont*g(p(revng,revnp))*(Z1^Z2^/(T^Z2^+Z1^Z2^))

**When light is off**

MnPo'=trPo*G-tmc*MnPo-umPo*MnPo

MnPt’=trPt*G-tmc*MnPt-umPt*MnPt

**Promoter Activity**

**E-box**

GR'=bin*(Sum[x[0][kk][0][1][1],(8)])*(1-G-GR)-unbin*GR G'=bin*x[0][0][0][1][1]*(1-G-GR)-unbin*G GrR'=binr*(Sum[x[0][kk][0][1][1],{kk,1,2}])*(1-Gr-GrR)-unbinr*GrR Gr'=binr*x[0][0][0][1][1]*(1-Gr-GrR)-unbinr*Gr GcR'=binc*(Sum[x[0][kk][0][1][1],{kk,1,2}])*(1-Gc-GcR)-unbinc*GcR Gc'=binc*x[0][0][0][1][1]*(1-Gc-GcR)-unbinc*Gc

**RORE**

GBR'=binrev*(revn+revng+revngp+revnp)*GB-unbinrev*GBR GB'=-binrev*(revn+revng+revngp+revnp)*GB+unbinrev*GBR

GBRb'=binrevb*(revn+revng+revngp+revnp)*GBb-unbinrevb*GBRb GBb'=-binrevb*(revn+revng+revngp+revnp)*GBb+unbinrevb*GBRb

**Transcription**

MnPo'=trPo*G-tmc*MnPo-umPo*MnPo McPo'=tmc*MnPo-umPo*McPo MnPt'=trPt*G-tmc*MnPt-umPt*MnPt McPt'=tmc*MnPt-umPt*McPt MnRt'=trRt*Gc-tmc*MnRt-umRt*MnRt McRt'=tmc*MnRt-umRt*McRt

MnRev'=trRev*x[0][0][0][1][1]*Gr-tmcrev*MnRev-umRev*MnRev McRev'=tmcrev*MnRev-umRev*McRev

MnRo'=trRo*G*GB-tmc*MnRo-umRo*MnRo McRo'=tmc*MnRo-umRo*McRo MnB'=trB*GBb-tmc*MnB-umB*MnB McB'=tmc*MnB-umB*McB

MnNp'=trNp*GB- tmc*MnNp - umNp*MnNp

McNp'=tmc*MnNp-umNp*McNp

**Secondary Feedback Loop**

B'=tlb*McB-cbin*B*Cl+uncbin*BC-ub*B Cl'=tlnp*McNp+tlc-cbin*B*Cl+uncbin*BC-uc*Cl BC'=cbin*B*Cl-uncbin*BC-phos*BC-ubc*BC

cyrev'=tlrev*McRev-(nlrev+urev)*cyrev-ag*cyrev*(x[0][0][2][0][0])+nerev*revn+dg*cyrevg revn'=-(nerev+urev)*revn-ag*Nf*revn*(x[0][0][2][1][0])+nlrev*cyrev+dg*(revng) cyrevg'=ag*cyrev*x[0][0][2][0][0]-(dg+gto+urev+nlrev)*cyrevg+nerev*revng revng'=ag*Nf*revn*x[0][0][2][1][0]-(dg+gto+urev+nerev)*revng+nlrev*cyrevg cyrevgp'=gto*cyrevg-(dg+uprev+nlrev)*cyrevgp+nerev*revngp

revngp'=gto*revng-(dg+uprev+nerev)*revngp+nlrev*cyrevgp cyrevp'=dg*(cyrevgp)-(uprev+nlrev)*cyrevp+nerev*revnp revnp'=dg*(revngp)-(uprev+nerev)*revnp+nlrev*cyrevp

**Translation**

x[j][k][l][m][n]'= If[(j=1)&&(k=0)&&(l=0)&&(m=0)&&(n=0),tlp*McPo,0]

+If[(j=3)&&(k=0)&&(l=0)&&(m=0)&&(n=0),tlp*McPt,0]

+If[(j=0)&&(k=1)&&(l=0)&&(m=0)&&(n=0),tlr*McRo,0]

+If[(j=0)&&(k=2)&&(l=0)&&(m=0)&&(n=0),tlr*McRt,0]

**Binding/Unbinding**

**CK1-CK1 inhibitor (PF-670462)**

[x[j][k][l][m][n]'=

If[(l==1)&&(m==0)&&(n==0),-inbin*Inh*x[j][k][l][m][n]+inubin*x[j][k][4][m][n],0]+

If[(l==3)&&(m==0)&&(n==0),-inbin*Inh*x[j][k][l][m][n]+inubin*x[j][k][5][m][n],0]+

If[(l==4)&&(m==0)&&(n==0),inbin*Inh*x[j][k][1][m][n]-inubin*x[j][k][l][m][n],0]+

If[(l==5)&&(m==0)&&(n==0),inbin*Inh*x[j][k][3][m][n]-inubin*x[j][k][l][m][n],0]+ If[(l==1)&&(m==1),-inbin*Nf*nInh*x[j][k][l][m][n]+

inubin*x[j][k][4][m][n],0]+

If[(l==3)&&(m==1),-inbin*Nf*nInh*x[j][k][l][m][n]+inubin*x[j][k][5][m][n],0]+

If[(l==4)&&(m==1),inbin*Nf*nInh*x[j][k][1][m][n]-inubin*x[j][k][l][m][n],0]+

If[(l==5)&&(m==1),inbin*Nf*nInh*x[j][k][3][m][n]-inubin*x[j][k][l][m][n],0]

**PER-CRY**

x[j][k][l][m][n]'=

If[(k==0)&&(n==0)&&((j==2)||(j==4)||(j==5)||(j==6)),-

ar*If[m==1,Nf,1]*x[j][k][l][m][n]*Sum[x[0][kk][0][m][0],{kk,1,2}]+dr*Sum[x[j][kk][l][m][n],{kk,1,2}],0]+ If[(j==0)&&((k==1)||(k==2))&&(l==0)&&(n==0),-

ar*If[m==1,Nf,1]*x[j][k][l][m][n]*Sum[x[jj][0][ll][m][0],{jj,{2,4,5,6}},{ll,0,5}]+dr*Sum[x[jj][k][ll][m][n],{jj,

{2,4,5,6}},{ll,0,5}],0]+

If[((j==2)||(j==4)||(j==5)||(j==6))&&((k==1)||(k==2))&&(n==0), ar*If[m==1,Nf,1]*x[0][k][0][m][n]*x[j][0

][l][m][0]- dr*x[j][k][l][m][n],0]+

If[(k==0)&&(n==1)&&((j==2)||(j==4)||(j==5)||(j==6))&&(m==1),-

ar*Nf*x[j][k][l][m][n]*Sum[x[0][kk][0][m][0],{kk,1,2}]+dr*Sum[x[j][kk][l][m][n],{kk,1,2}],0]+ If[(j==0)&&((k==1)||(k==2))&&(l==0)&&(m==1)&&(n==0),-

ar*Nf*x[j][k][l][m][n]*Sum[x[jj][0][ll][m][1],{jj,{2,4,5,6}},{ll,0,5}]+dr*Sum[x[jj][k][ll][m][1],{jj,{2,4,5,6}},{ll

,0,5}],0]+

If[((j==2)||(j==4)||(j==5)||(j==6))&&((k==1)||(k== 2))&&(m==1)&&(n==1), ar*Nf*x[j][0][l][m][n]*x[0][k][ 0][m][0]-dr*x[j][k][l][m][n],0]+

If[(k==0)&&(n==0)&&((j==2)||(j==4)||(j==5)||(j==6))&&(m==1),-

ar*Nf*x[j][k][l][m][n]*Sum[x[0][kk][0][m][1],{kk,1,2}]+dr*Sum[x[j][kk][l][m][1],{kk,1,2}],0]+ If[(j==0)&&((k==1)||(k==2))&&(l==0)&&(m==1)&&(n==1),-

ar*Nf*x[j][k][l][m][n]* Sum[x[jj][0][ll][m][0],{jj,{2,4,5,6}},{ll,0,5}]+dr*Sum[x[jj][k][ll][m][n],{jj,{2,4,5,6}},{ll

,0,5}],0]+

If[((j==2)||(j==4)||(j==5)||(j==6))&&((k==1)||(k==2))&&(m==1)&&(n==1), ar*Nf*x[j][0][l][m][0]*x[0][k][ 0][m][1]-dr*x[j][k][l][m][n],0]

**PER-CKI**

x[j][k][l][m][n]'=

If[(l==0)&&(j>0)&&(n==0),-

ac*If[m==1,Nf,1]*x[j][k][l][m][n]*Sum[x[0][0][ll][m][0],{ll,1,4,3}]+dc*Sum[x[j][k][ll][m][n],{ll,1,4,3}],0]+ If[(j==0)&&(k==0)&&((l==1)||(l==4))&&(n==0),-

ac*If[m==1,Nf,1]*x[j][k][l][m][n]*Sum[x[jj][kk][0][m][0],{jj,1,6},{kk,0,2}]+dc*Sum[x[jj][kk][l][m][0],{jj,1

,6},{kk,0,2}],0]+

If[(j>0)&&((l==1)||(l==4))&&(n==0),ac*If[m==1,Nf,1]*x[0][0][l][m][0]*x[j][k][0][m][n]- dc*x[j][k][l][m][n],0]+

If[(l==0)&&(j>0)&&(m==1)&&(n==1),-

ac*Nf*x[j][k][l][m][n]*Sum[x[0][0][ll][m][0],{ll,1,4,3}]+dc*Sum[x[j][k][ll][m][n],{ll,1,4,3}],0]+ If[(j==0)&&(k==0)&&((l==1)||(l==4))&&(m==1)&&(n==0),-

ac*Nf*x[j][k][l][m][n]*Sum[x[jj][kk][0][m][1],{jj,1,6},{kk,0,2}]+dc*Sum[x[jj][kk][l][m][1],{jj,1,6},{kk,0,2}],0]+

If[(j>0)&&((l==1)||(l==4))&&(m==1)&&(n==1),ac*Nf*x[0][0][l][m][0]*x[j][k][0][m][n]- dc*x[j][k][l][m][n], 0]+If[(j>2)&&(l==2)&&(n==0),-ac*If[m==1,Nf,1]* x[j][k][l][m][n]*x[0][0][1][m][0]+dc*x[j][k][3][m][n],0]+ If[(j==0)&&(k==0)&&(l==1)&&(n==0),-

ac*If[m==1,Nf,1]* x[j][k][l][m][n]*Sum[x[jj][kk][2][m][0],{jj,3,6},{kk,0,2}]+dc*Sum[x[jj][kk][3][m][0],{jj,3

,6},{kk,0,2}],0]+If[(j>2)&&(l==3)&&(n==0),ac*If[m==1,Nf,1]*x[j][k][2][m][n]*x[0][0][1][m][0]-

dc*x[j][k][l][m][n],0]+

If[(j>2)&&(l==2)&&(n==0),-ac*If[m==1,Nf,1]*x[j][k][l][m][n]*x[0][0][4][m][0]+dc*x[j][k][5][m][n],0]+ If[(j==0)&&(k==0)&&(l==4)&&(n==0),-

ac*If[m==1,Nf,1]*x[j][k][l][m][n]*Sum[x[jj][kk][2][m][0],{jj,3,6},{kk,0,2}]+dc*Sum[x[jj][kk][5][m][0],{jj,3

,6},{kk,0,2}],0]+

If[(j>2)&&(l==5)&&(n==0),ac*If[m==1,Nf,1]*x[j][k][2][m][n]*x[0][0][4][m][0]-dc*x[j][k][l][m][n],0]+

If[(l==2)&&(j>2)&&(m==1)&&(n==1),-ac*Nf*x[j][k][l][m][n]*x[0][0][1][m][0]+dc*x[j][k][3][m][n],0]+ If[(j==0)&&(k==0)&&(l==1)&&(m==1)&&(n==0),-

ac*Nf* x[j][k][l][m][n]*Sum[x[jj][kk][2][m][1],{jj,3,6},{kk,0,2}]+dc*Sum[x[jj][kk][3][m][1],{jj,3,6},{kk,0,2}

],0]+

If[(j>2)&&(l==3)&&(m==1)&&(n==1),ac*Nf*x[0][0][1][m][0]*x[j][k][2][m][n]-dc*x[j][k][l][m][n],0]+

If[(l==2)&&(j>2)&&(m==1)&&(n==1),-ac*Nf*x[j][k][l][m][n]*x[0][0][4][m][0]+dc*x[j][k][5][m][n],0]+ If[(j==0)&&(k==0)&&(l==4)&&(m==1)&&(n==0),-

ac*Nf*x[j][j][l][m][n]*Sum[x[jj][kk][2][m][1],{jj,3,6},{kk,0,2}]+dc*Sum[x[jj][kk][5][m][1],{jj,3,6},{kk,0,2}]

,0]+

If[(j>2)&&(l==5)&&(m==1)&&(n==1),ac*Nf*x[0][0][4][m][0]*x[j][k][2][m][n]-dc*x[j][k][l][m][n],0]

**PER-GSK3β**

x[j][k][l][m][n]'=

If[(j>2)&&((l==0)||(l==1)),-If[m==1,Nf,1]*agp*x[j][k][l][m][n]*x[0][0][2][m][0]+dg*x[j][k][l+2][m][n],0]+

If[(j>2)&&(l==4),-If[m==1,Nf,1]*agp*x[j][k][l][m][n]*x[0][0][2][m][0]+dg*x[j][k][5][m][n],0]+

If[(j>2)&&((l==2)||(l==3)),If[m==1,Nf,1]*agp*x[j][k][l-2][m][n]*x[0][0][2][m][0]-dg*x[j][k][l][m][n],0]+

If[(j>2)&&(l==5),If[m==1,Nf,1]*agp*x[j][k][4][m][n]*x[0][0][2][m][0]-dg*x[j][k][l][m][n],0]+

If[(j==0)&&(k==0)&&(l==2)&&(n==0),-

If[m==1,Nf,1]*agp* Sum[x[jj][kk][ll][m][nn],{jj,3,6},{kk,0,2},{ll,{0,1,4}},{nn, 0,1}]*x[j][k][l][m][n]+ dg*Su

m[ x[jj][kk][ll][m][nn],{jj,3,6},{kk,0,2},{ll,{2,3,5}},{nn,0,1}],0]

**PER-BMALs-CLOCK/NPAS2**

x[j][k][l][m][n]'=

If[(j>0)&&(m==1)&&(n==0),-bbin*Nf*x[j][k][l][m][n]*x[0][0][0][m][1]+unbbin*x[j][k][l][m][1],0]+ If[(j==0)&&(k==0)&&(n>0)&&(l==0)&&(m==1),-

bbin*Nf* x[0][0][0][m][n]*Sum[x[jj][kk][ll][m][0],{jj,1,6},{kk,0,2},{ll,0,5}]+unbbin*Sum[x[jj][kk][ll][m][n]

,{jj,1,6},{kk,0,2},{ll,0,5}],0]+If[(j>0)&&(m==1)&&(n>0),bbin*Nf*x[j][k][l][m][0]*x[0][0][0][m][n]-

unbbin*x[j][k][l][m][n],0]

**CRY-BMALs-CLOCK/NPAS2**

x[j][k][l][m][n]'=

If[(j=0)&&(k>0)&&(l=0)&&(m=1)&&(n=0),- cbbin*Nf*x[j][k][l][m][n]*x[0][0][0][m][1]+uncbbin*x[j][k][l][m][1],0]+ If[(j=0)&&(k=0)&&(l=0)&&(m=1)&&(n=1),- cbbin*Nf*Sum[x[0][kk][0][m][0],{kk,1,2}]*x[j][k][l][m][n]+uncbbin*Sum[x[0][kk][0][m][n],{kk,1,2}],0]+

If[(j=0)&&(k>0)&&(l=0)&&(m=1)&&(n=1),cbbin*Nf*x[j][k][l][m][0]*x[0][0][0][m][n]- uncbbin*x[j][k][l][m][n],0]

**REV-ERBs-GSK3β**

x[j][k][l][m][n]'=

If[(j=0)&&(k=0)&&(l=2)&&(m=0)&&(n=0),-ag*cyrev*x[j][k][l][m][n]+(dg)*cyrevg+(dg)*cyrevgp,0]+

If[(j=0)&&(k=0)&&(l=2)&&(m=1)&&(n=0),-ag*Nf*revn*x[j][k][l][m][n]+(dg)*revng+(dg)*revngp,0]

**Translocation**

**PER binding proteins**

x[j][k][l][m][n]'=

If[((j=2)||(j=4)||(j=5)||(j=6))&&(m=1),-ne*If[(n=0),1,0]*x[j][k][l][m][n]+If[(n=0),1,0]*nl*x[j][k][l][0][n],0]+

If[((j=2)||(j=4)||(j=5)||(j=6))&&(m=0),ne*If[(n=0),1,0]*x[j][k][l][1][n]-If[(n=0),1,0]*nl*x[j][k][l][m][n],0]+

**BMALs-CLOCK/NPAS2**

x[j][k][l][m][n]'=

If[(j=0)&&(k=0)&&(l=0)&&(m=1)&&(n=1,nlbc*x[j][k][l][0][n],0]+

If[(j=0)&&(k=0)&&(l=0)&&(m=0)&&(n=1),-nlbc*x[j][k][l][m][n],0]+

**CK1 and GSK3b Kinase**

x[j][k][l][m][n]'=

If[(j=0)&&(k=0)&&((l=1)||(l=2))&&(m=1)&&(n=0),-lne*x[j][k][l][m][n],0]+

If[(j=0)&&(k=0)&&((l=1)||(l=2))&&(m=0)&&(n=0),lne*x[j][k][l][1][n],0]

**CK1 inhibitor (PF-670462)**

x[j][k][l][m][n]'=

If[(j==0)&&(k==0)&&((l==4))&&(m==1)&&(n==0),-lnei* x[j][k][l][m][n],0]+

If[(j==0)&&(k==0)&&((l==4))&&(m==0)&&(n==0),lnei*x[j][k][l][1][n],0]

**Phosphorylation**

x[j][k][l][m][n]'=

If[((j=1))&&(l=1)&&(k=0)&&(m=0)&&(n=0),-hoo*x[j][k][l][m][n],0]+

If[((j=2))&&(l=1)&&(k=0)&&(m=0)&&(n=0),+hoo*x[1][k][l][m][n],0]+

If[((j=3)||(j=5))&&((l=1)||(l=3))&&(k=0),-hto*x[j][k][l][m][n],0]+

If[((j=4)||(j=6))&&((l=1)||(l=3))&&(k=0),hto*x[j-1][k][l][m][n],0]+ If[((j=3)||(j=4))&&((l=2)||(l=3)),-gto*x[j][k][l][m][n],0]+If[((j=5)||(j=6))&&((l=2)||(l=3)),gto*x[j- 2][k][l][m][n],0]+If[(j=0)&&(k=0)&&(l=0)&&(m=0)&&(n=1),phos*BC,0]+

**Degradation**

**CRY**

x[j][k][l][m][n]'=

If[(j=0)&&(k=1)&&(l=0)&&(n=0),-uro*x[j][k][l][m][n],0]+

If[(j=0)&&(k=2)&&(l=0)&&(n=0),-urt*x[j][k][l][m][n],0]+

If[(j=0)&&(k=1)&&(l=0)&&(m=1)&&(n=1),-uro*x[j][k][l][m][n],0]+

If[(j=0)&&(k=2)&&(l=0)&&(m=1)&&(n=1),-urt*x[j][k][l][m][n],0]+

**PER**

x[j][k][l][m][n]'=

If[((j==1)||(j==3)||(j==5))&&(k==0),-If[(m==0)&&(n==1),0,1]*upu*x[j][k][l][m][n],0]+

If[((j==2))&&(k==0),-If[(m==0)&&(n==1),0,1]*up* x[j][k][l][m][n],0]+

If[((j==4)||(j==6))&&(k==0),-If[(m==0)&&(n==1),0,1]*up*x[j][k][l][m][n],0]+

If[(j==0)&&(k==0)&&(l==1)&&(n==0),up*Sum[x[jj][0][ll][m][nn],{jj,4,6,2},{nn,0,1},{ll,1,3,2}]+up*Sum [x[jj][0][ll][m][nn],{jj,2,2},{nn,0,1},{ll,1,3,2}]+upu*Sum[x[jj][0][ll][m][nn],{jj,1,5,2},{nn,0,1},{ll,1,3,2}],0]+

If[(j==0)&&(k==0)&&(l==4)&&(n==0),up*Sum[x[jj][0][ll][m][nn],{jj,4,6,2},{nn,0,1},{ll,4,5}]+up*Sum[x[jj][0][ll][m][nn],{jj,2,2},{nn,0,1},{ll,4,5}]+upu*Sum[x[jj][0][ll][m][nn],{jj,1,5,2},{nn,0,1},{ll,4,5}],0]+

If[(j==0)&&(k==0)&&(l==2)&&(n==0), up*Sum[x[jj][0][ll][m][nn],{jj,4,6,2},{ll,{2,3,5}},{nn,0,1}]+up*Sum

[x[jj][0][ll][m][nn],{jj,2,2},{ll,{2,3,5}},{nn,0,1}]+upu*Sum[x[jj][0][ll][m][nn],{jj,1,5,2},{ll,{2,3,5}},{nn,0,1}],0]+

If[(j==0)&&(k==0)&&(l==0)&&(n==1)&&(m==1),up*Sum[x[jj][0][ll][m][n],{jj,4,6,2},{ll,0,5}]+up*Sum

[x[jj][0][ll][m][n],{jj,2,2},{ll,0,5}]+upu*Sum[x[jj][0][ll][m][n],{jj,1,5,2},{ll,0,5}],0]

**BMALs-CLOCK/NPAS2**

x[j][k][l][m][n]'=

If[(j>0)&&(k=0)&&(m=1)&&(n=1),-ubc*x[j][k][l][m][n],0]+

If[(j=0)&&(k=0)&&(l=0)&&(n=1),-ubc*x[j][k][l][m][n],0]+

If[(j>0)&&(k=0)&&(m=1)&&(n=0),ubc*x[j][k][l][m][1],0]

**REV-ERBs**

x[j][k][l][m][n]'= If[(j=0)&&(k=0)&&(l=2)&&(m=0)&&(n=0),urev*cyrevg+uprev*cyrevgp,0]+ If[(j=0)&&(k=0)&&(l=2)&&(m=1)&&(n=0),urev*revng+uprev*revngp,0]

**Transcriptional Activity of GSK3**β

gto'=trgto*G*GB-ugto*gto

**Reference**

Kim JK, Forger DB, Marconi M, Wood D, Doran A, Wager T, Chang C, Walton KM (2013) Modeling and validating chronic pharmacological manipulation of circadian rhythms. *CPT Pharmacometrics Syst Pharmacol* **2:** e57
