# Supplementary material for: Systems approach reveals photosensitivity and PER2 level as determinants of clock‐modulator efficacy
Source: Mol Syst Biol. 2019 Jul 8;15(7):e8838. doi: 10.15252/msb.20198838 (PMC6613017; doi:10.15252/msb.20198838)
Supplement: Supplementary file 2 — Expanded View Figures PDF [file MSB-15-e8838-s002.pdf]

Expanded View Figures

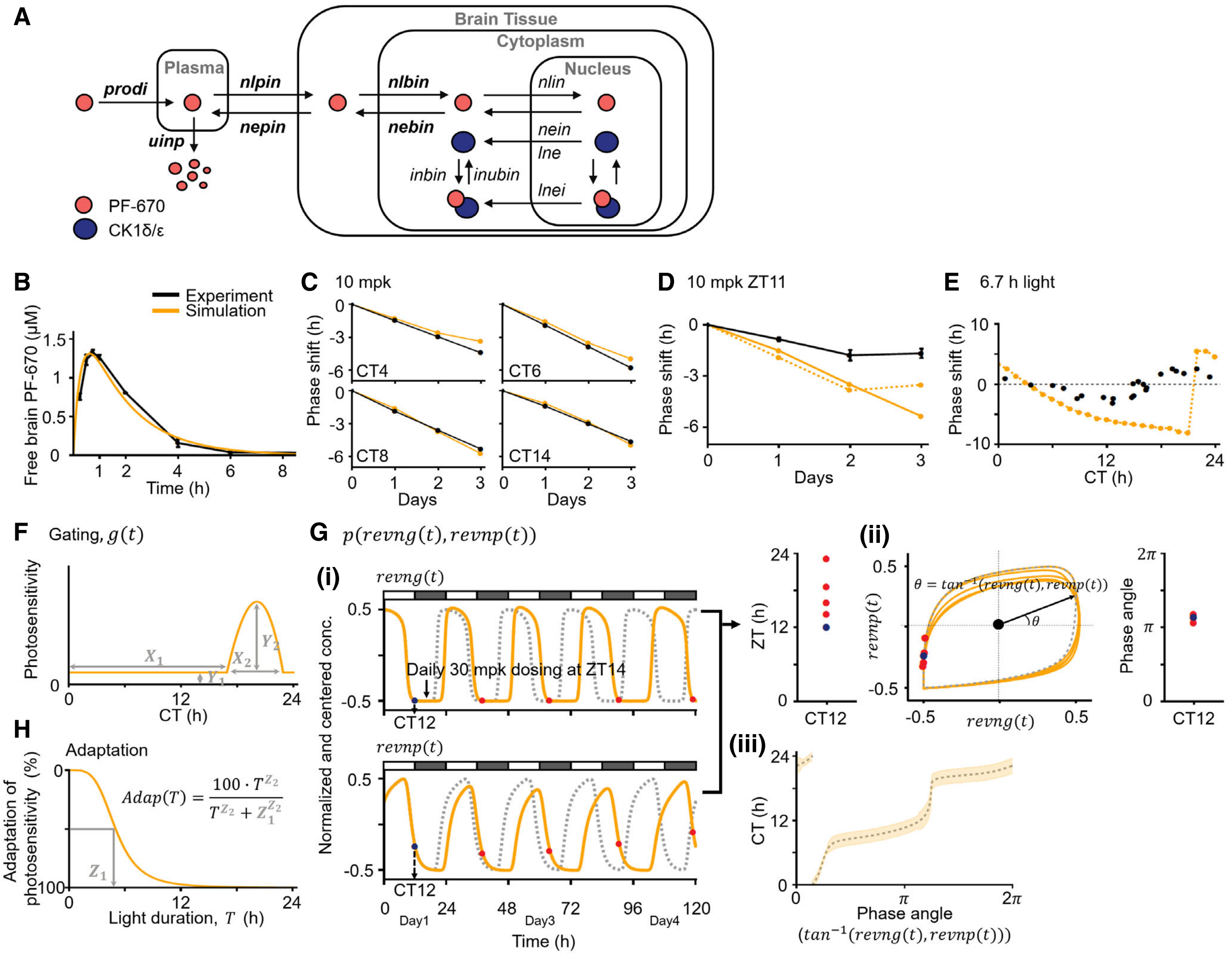

Figure EV1.

Figure EV1. The original model (Kim *et al*, 2013) with modified pharmacokinetic parameters accurately captures DD dosing but not LD dosing in NHPs.

A Model diagram describing the dynamics of PF-670, which is modified from (Kim *et al*, 2013). As was done in the original model (Kim *et al*, 2013), due to the fast absorption of PF-670 into the brain tissue of NHPs ($t_{max} < 0.75$ h; Fig 1B), we assume that PF-670 directly comes into the plasma compartment with the initial concentration, *prodi*, which is cleared with the rate of *uinp*. Furthermore, the transfer rate of PF-670 from plasma to brain tissue (nlpin) is assumed to be equal to that from brain tissue to plasma (nepin) as free brain and plasma PF-670 concentrations are predicted to be equal by physiologically based PK modeling (see Materials and Methods for details). After entering the brain tissue compartment, PF-670 transfers between brain tissue and cytoplasm with the rates of *nlbin* and *nebin*, respectively. PF-670 then traffics between the cytoplasm and nucleus with the rates of *nlin* and *nein*, respectively. In a cell, PF-670 and CK1δ/ε form a reversible complex with the binding rate, *inbin,* and unbinding rate, *inubin*. The rate of nuclear export of CK1δ/ε bound to PF-670 (*lnei*) is allowed to be different from the rate of nuclear export of CK1δ/ε unbound to PF-670 (*lne*) following the original model (Kim *et al*, 2013).

B, C While the six pharmacodynamic parameters were taken from the original model (Kim *et al*, 2013), the six PK parameters, highlighted as bold in (A), were modified to reproduce the free PF-670 exposure in the brain tissue of NHPs ($n = 2$; mean ± SEM; B) and its DD dosing effects in NHPs at various initial dosing times (CT 4–14 h) (C). See Dataset EV1 for the values of the modified PK parameters. Here, the PF-670-induced phase delay from which the vehicle-induced phase shift is subtracted was used (Fig 1E).

D, E Unlike DD dosing (C), the model overestimates the phase delay induced by 3-day 10 mpk LD dosing ($n = 8$; mean ± SEM; Fig 1F) (solid line; D). On the other hand, when the light-induced Per1/2 gene transcription is increased to reduce the simulated phase delay (dashed line; D), a 6.7-h light pulse leads to an unrealistically large phase shift in the model (~10 h) compared with the experimentally measured one in humans (black dots; E) adopted from (Khalsa *et al*, 2003). Here, to get the high photosensitivity (dashed line; D and E), the values of parameters describing light-induced Per1/2 transcription rates (*lono/lont*; Dataset EV1) are increased threefold from the values used in the original model (Kim *et al*, 2013).

F To accurately capture the effect of LD dosing in NHPs, gating for light, which is denoted by function *g* in Materials and Methods and Appendix Equation S1, is incorporated into the model (Fig 2Aii). The shape of the gating is determined by four parameters: $\mathbf{X_1}$ determines the circadian time when the gating becomes weaker and thus the photosensitivity increases. $\mathbf{X_2}$ describes the range of high photosensitivity zones. $\mathbf{Y_1}$ describes the photosensitivity of the circadian clock when it is fully inhibited, which is assumed to be constant for simplicity. $\mathbf{Y_2}$ describes the maximum photosensitivity of the circadian clock. To connect these photo-insensitive and photosensitive zones continuously, a piecewise polynomial interpolation is used (see Code EV1). Note that the gating depends on the CT.

G To estimate the input CT for gating (F) even when the circadian phase is altered by a stimulus (i.e., light and PF-670), we constructed the function *p*, which estimates the CT from the phase angle of limit cycle of two clock variables, *revng* and *revnp* (Table EV1). (i) When the circadian clock is entrained by external light (i.e., LD 12:12), the CT can be simply approximated by ZT (e.g., CT12≈ZT12; blue circle). However, if the circadian phase is delayed by PF-670, ZT, which corresponds to the same CT (e.g., CT12), changes dramatically (e.g., ZT14, 16, 19, and 23; red circles). (ii) On the other hand, the phase angles of limit cycle of *revng* and *revnp*, which corresponds to the same CT, change little (e.g., blue and red circles corresponding to CT12 in (i)). The concentration of *revng* and *revnp* is normalized by their maximum concentration in LD 12:12 and is centered by the average of their maximum and minimum concentration. (iii) Based on this feature, we constructed the function p, which is the function of the phase angle of the limit cycle (i.e., $\tan^{-1}$ (*revng*(*t*), *revnp*(*t*)) for CT when the model is entrained to LD 12:12 (gray dashed line). This allows the model to accurately predict the CT from the phase angle even when the circadian phase is altered by PF-670 (orange range). The orange range represents the mean ± SD of the predicted CTs using the phase angle when a single daily 30 mpk dosing is given at ZT14 for 20 days. Note that *p* is accurate up to a considerably high dose (~80 mpk) as the limit cycle is stable.

H The adaptation for light is incorporated into the model (Fig 2Aii). The shape of adaptation is described with a Hill function with two parameters: $\mathbf{Z_1}$ and $\mathbf{Z_2}$ determine the light duration which reduces the photosensitivity by 50% and how sharply the photosensitivity decreases, respectively.

Figure EV2. Filtering procedure to identify accurate gating and adaptation for light in the model.

A 911 pairs of gating and adaptation, with which the model accurately simulates the 3-day LD dosing effect in NHPs ($n = 8$; mean ± SEM; Fig 1F) (left) and the magnitude of human PRC to a 6.7-h light pulse (Khalsa *et al*, 2003), were found using the simulated annealing method (see Materials and Methods for details). The photosensitivity is normalized so that the maximum photosensitivity of initially estimated gatings becomes unified (center). 100% adaptation in photosensitivity means that Per1/2 gene transcription is no longer induced by light (right).

B 152 pairs of gating and adaptation with which simulated PRCs to a 12-h light pulse are discontinuous were filtered out (left), which is caused by the slow adaptation exaggerating the light effect (right).

C 323 pairs of gating and adaptation were filtered out with which simulated light PRCs to a 6.7-h light pulse have a too large phase shift between CT0 and CT6 (left) due to overestimated photosensitivity (center). Here, the human PRC to a 6.7-h light pulse is adopted from (Khalsa *et al*, 2003).

D 327 pairs of gating and adaptation were filtered out with which the simulated PRCs to 3-cycle 5-h light pulses (left) have either a too large phase shift due to overestimated photosensitivity between CT6 and CT12 (center) or an abrupt jump between CT16 and CT20 due to an underestimated range of high photosensitivity zone (center). Here, the human PRC to 3-cycle 5-h light pulses is adopted from (Khalsa *et al*, 1997).

E 99 pairs of gating and adaptation with which the model fails to simulate the human PRC to a 6.7-h light pulse (Khalsa *et al*, 2003) between CT15 and CT18 were filtered out (left).

F 10 pairs of gating and adaptation passed the filtering procedure. See Dataset EV2 for the values of the parameters describing the 10 pairs of gating and adaptation.

G, H The model with these 10 pairs of gating and adaptation (F) accurately simulates the human PRC to a 6.7-h light pulse (left) and 3-cycle 5-h light pulses (right) (G). The model accurately predicts the human PRC to a 3-h light pulse adopted from (Minors *et al*, 1991), which is not used in the estimation process (H).

 © 2019 The Authors

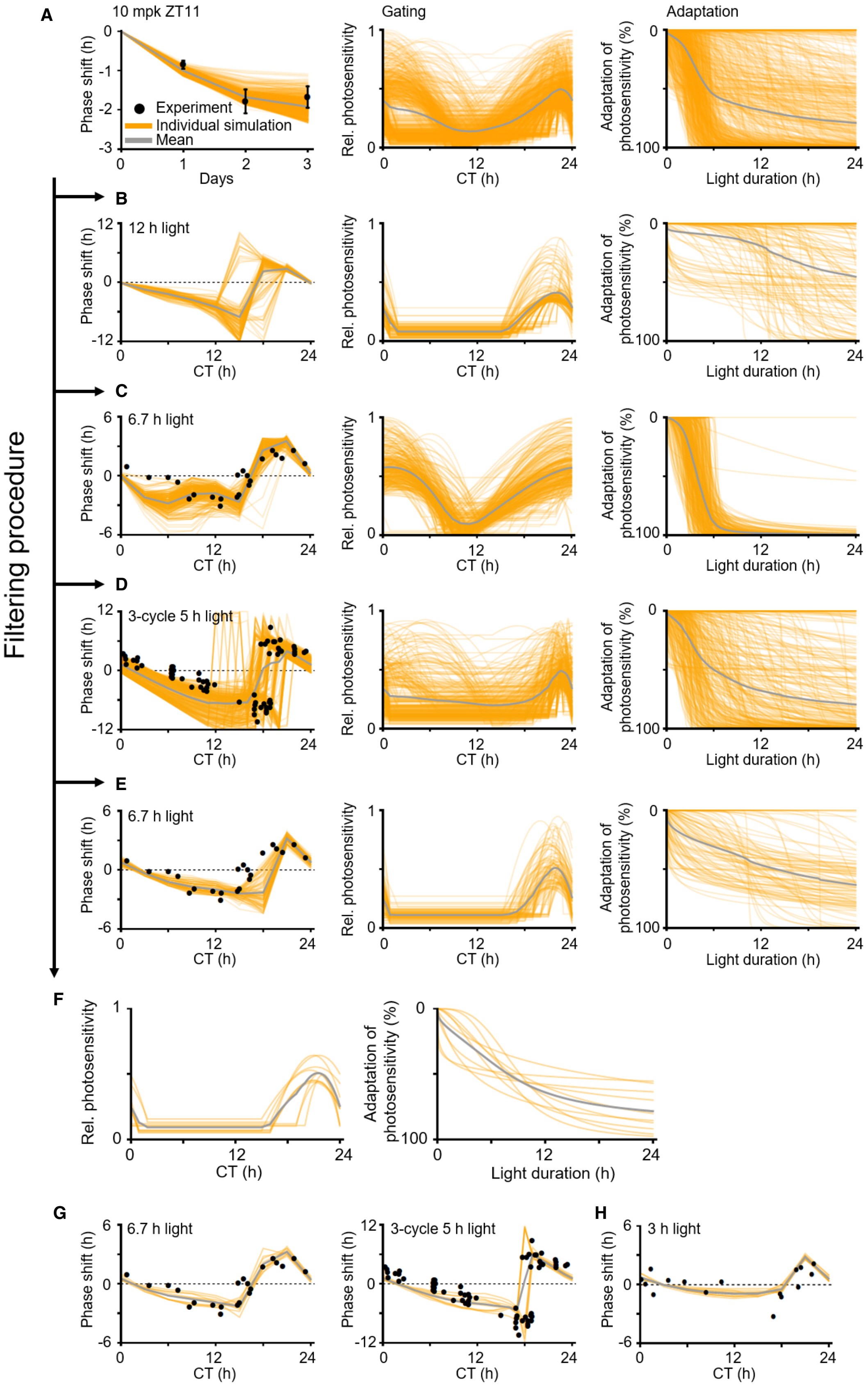
A
10 mpk ZT11
Experiment
Individual simulation
Mean
Phase shift (h)
Days
Gating
Rel. photosensitivity
CT (h)
Adaptation
Adaptation of photosensitivity (%)
Light duration (h)
Filtering procedure
B
12 h light
C
6.7 h light
D
3-cycle 5 h light
E
6.7 h light
F
G
6.7 h light
3-cycle 5 h light
H
3 h light

Figure EV2.

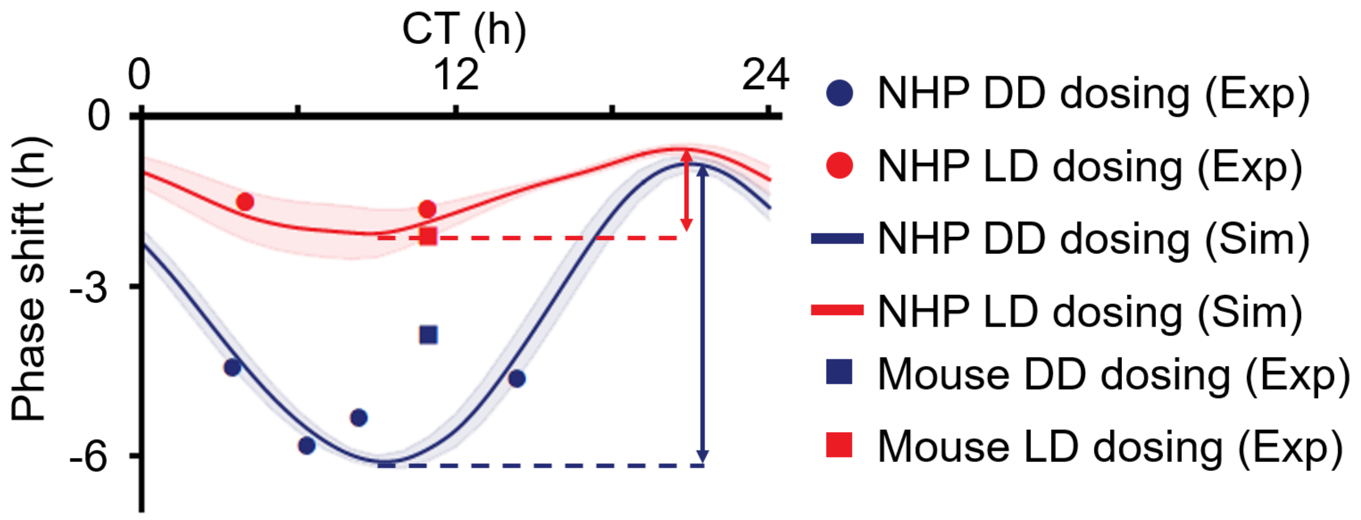

Figure EV3. The phase delays induced by 3-day PF-670 dosing in NHPs and mice under DD and LD.

NHPs and mice were treated with PF-670 for 3 days (Figs 1E and F, and 2F, and EV1C). Due to the higher drug exposure in NHPs than in mice (Fig 1B), all NHPs, which were treated with 10 mpk PF-670 at various dosing times (CT4, 6, 8, 14) in DD, show a larger phase delay (filled blue circles) than mice dosed with 32 mpk PF-670 at CT11 in DD (empty blue circle). Thus, the phase delay of NHPs induced by 10 mpk PF-670 dosing at CT11 is expected to be larger than that of mice dosed with 32 mpk PF-670 at CT11 under DD, which is supported by the model simulation (blue line). Due to the strong attenuation of the PF-670 effect by light in NHPs, the change in drug effect upon dosing time is much smaller in LD (from 0.6 h to 2.1 h; red arrow) than in DD (from 0.8 h to 6.1 h; blue arrow). Dosing time is denoted by the *x*-axis. The line and colored range represent the mean ± SD of the simulated phase delays of NHP models with the 10 pairs of gating and adaptation (Figs 2Aii and EV2F).

 © 2019 The Authors

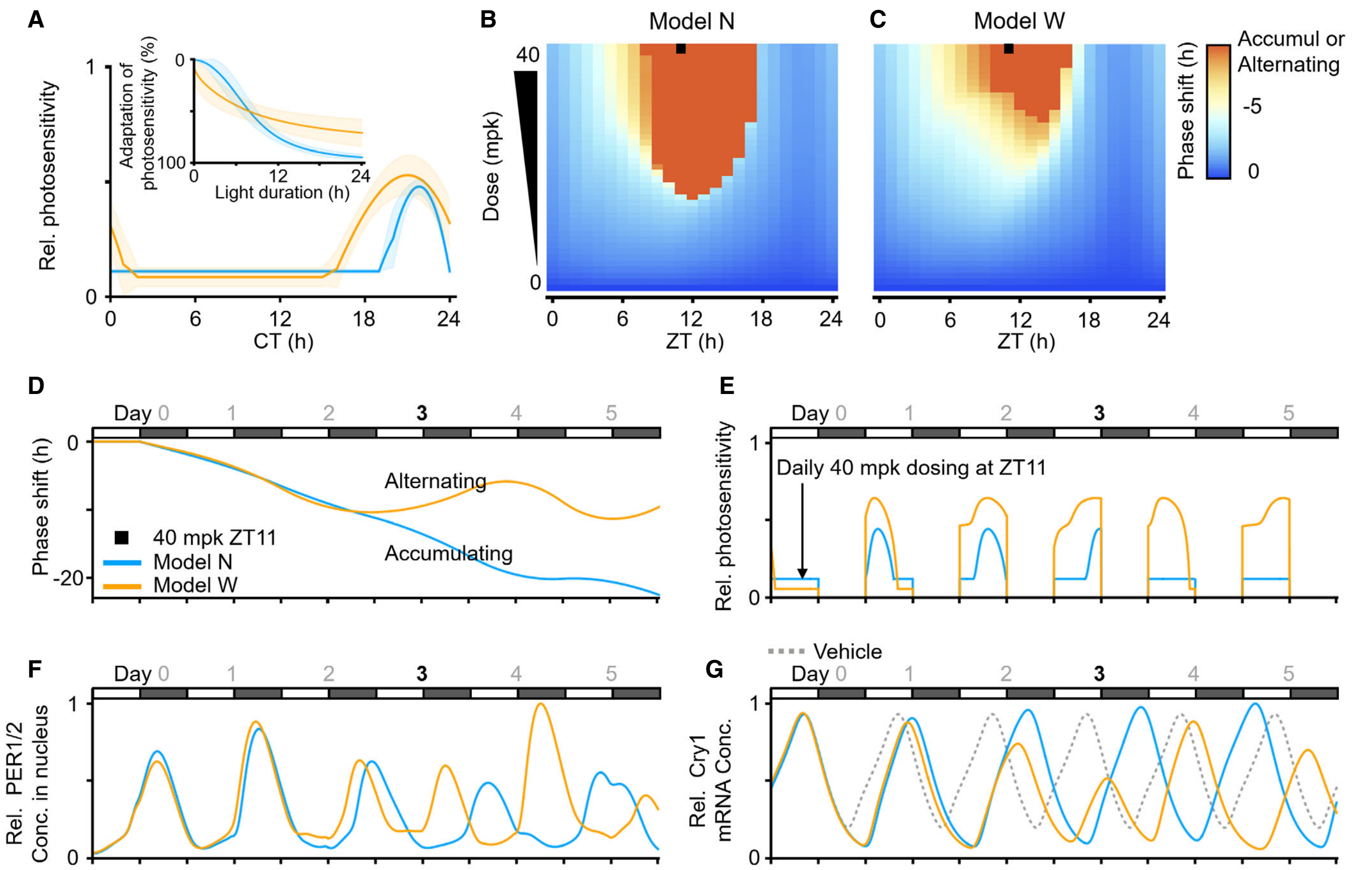

Figure EV4. The effect of PF-670 highly depends on the individual photosensitivity.

A The models with 10 pairs of gating and adaptation for light in Fig EV2F are divided into two classes: the models with gatings having a narrow (blue) or wide (orange) range of high photosensitivity. Among each class, we chose a representative model: The representative model with the gating having a narrow (wide) range of high photosensitivity zone is referred as model N (W) (see Dataset EV2 for details). The line and colored range represent the mean ± SD of the gatings and adaptations of each class, respectively.

B, C The phase delay induced by LD single daily dosing for 27 days with various dose levels and dosing times was simulated. Overall, model N (B) simulates a larger phase delay than model W (C) due to the weaker attenuating effect of light (Fig 3E). Here, LD 12:12 is used.

D A single daily 40 mpk dosing at ZT11 (black square at B) leads to a continually accumulating phase delay in model N as the dosing overcomes the attenuating effect of light, unlike a low dose (Fig 3F). On the other hand, despite the same regimen (black square at C), model W simulates an unstable alternating phase shift between delay and advance from day 3.

E–G Such an alternating phase shift in model W is due to a high dose-induced amplitude suppression. When the single daily dosing delays the circadian phase by ~10 h, the circadian time during the daytime corresponds to the time when photosensitivity is high in model W (CT14-CT2) (A and day 3; E). This prevents the decrease in the levels of PER1/2 proteins during the falling phase (daytime at day 3; F). Furthermore, the increase in the level of PER1/2 proteins is enhanced when PF-670 is dosed at ZT11 as it stabilizes PER1/2 protein. Due to this synergistic effect between light and PF-670, the level of PER1/2 protein in model W greatly increases (nighttime at day 3; F) compared with model N, which leads to a strong repression of the transcription for clock genes such as Cry1 mRNA and thus the amplitude suppression at day 3 unlike in model N (day 3; G). This amplitude suppression causes the circadian rhythms to reach a singular point (Diekman & Bose, 2018) and thus leads to an alternating phase shift (D). The vehicle in (G) represents a mean of relative Cry1 mRNA abundances simulated with models N and W.

© 2019 The Authors

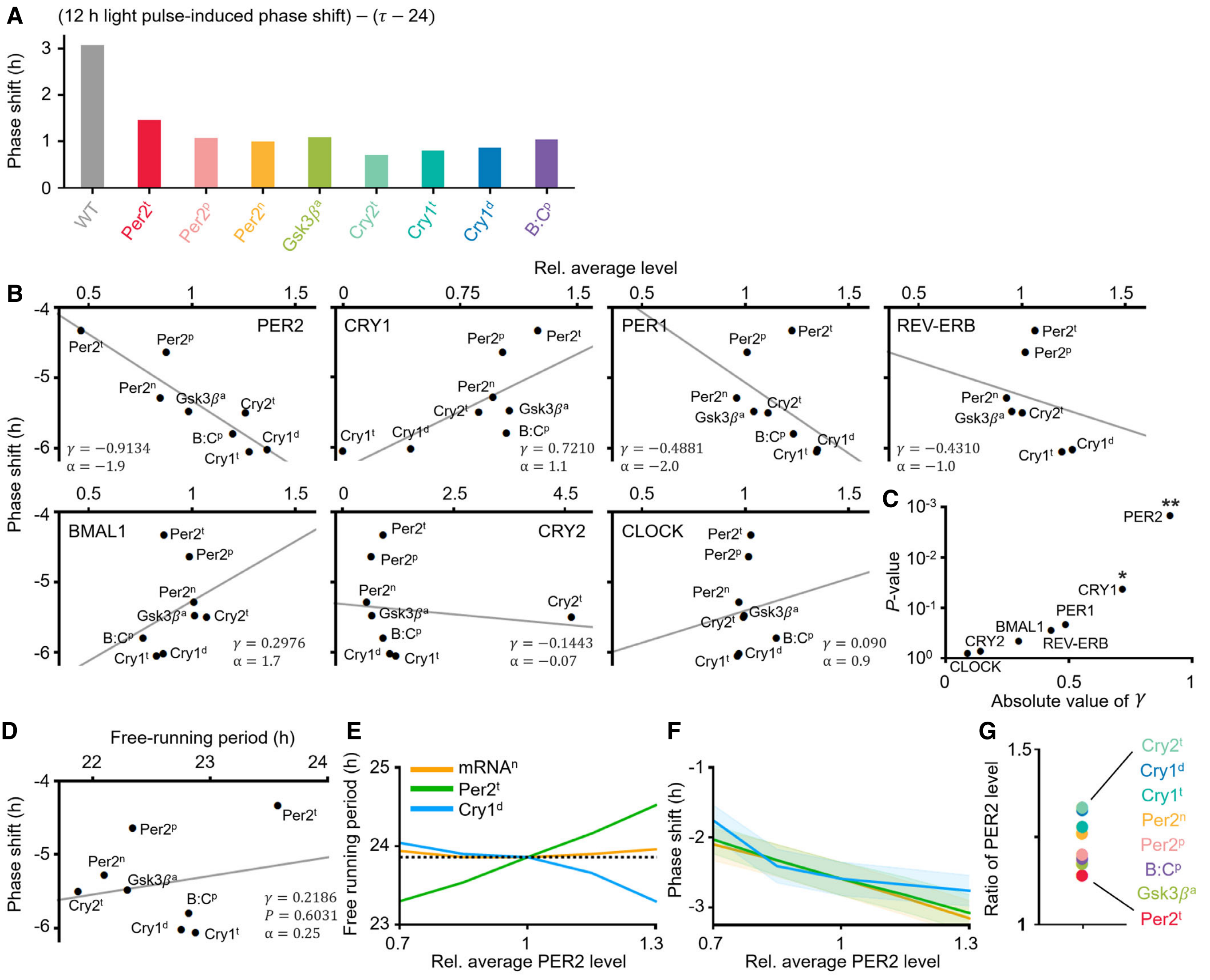

Figure EV5. The response of the ASPD models to light and PF-670: PER2 level is strongly correlated with the effect of CK1i.

A All ASPD models simulate the weaker light-induced attenuation of the effect of PF-670 than the WT model when PF-670 induces the 4-h phase delay under LD 12:12. As the free-running period (τ) of the ASPD models is shorter than 24 h (Fig 4A), the light needs to induce the phase shift (24-τ) to equalize the period of ASPD models to 24 h. Thus, by subtracting 24-τ from a light-induced phase shift to remove the entraining effect of light, the light-induced attenuation of the effect of PF-670 is estimated. The light-induced phase shift is simulated using a 12-h light pulse given at CT20 since it corresponds to the light pulse in LD 12:12 when PF-670 induces a 4-h phase delay. See Datasets EV2 and EV3 and Materials and Methods for details of the WT model and ASPD models.

B The correlation between the effect of PF-670 (red squares; Fig 4B) and the average level of various core clock proteins of the ASPD models (Dataset EV3). The line represents the least-square fitting line. r and α denote the Pearson's correlation coefficient and the slope of the least-square fitting line.

C The average protein level of PER2 is significantly more strongly correlated with the effect of PF-670 than that of other clock proteins. * and ** indicates $P < 0.05$ and $P < 0.001$, respectively. Here, *P*-value is estimated by Pearson's correlation test.

D The correlation between the effect of PF-670 (red squares; Fig 4B) and the free-running periods of the ASPD models (Fig 4A) is weak and not significant.

E, F As PER2 level increases in the model, regardless of the change in the free-running period (E), the effect of CK1i becomes stronger (F). The $mRNA^n$ (orange line), $Per2^t$ (green line), and $Cry1^d$ (blue line) were simulated by perturbing the parameters *tmc*, *trPt*, and *uro*, which describe the nuclear export rate of mRNA, the transcription rate for Per2, and the degradation rate for CRY1, respectively (see Dataset EV1 for details). The line and colored range in F represent the mean ± SEM of the phase delays induced by a single 20 mpk DD dosing at CT1, 2, 3, . . ., 24.

G The ratio of average PER2 levels in LD 8:16 and LD 16:8 when dosing occurs (red squares; Fig 4B) is higher than 1 in all ASPD models: PER2 level is higher in LD 8:16 than in LD16:8 when dosing occurs.

 © 2019 The Authors

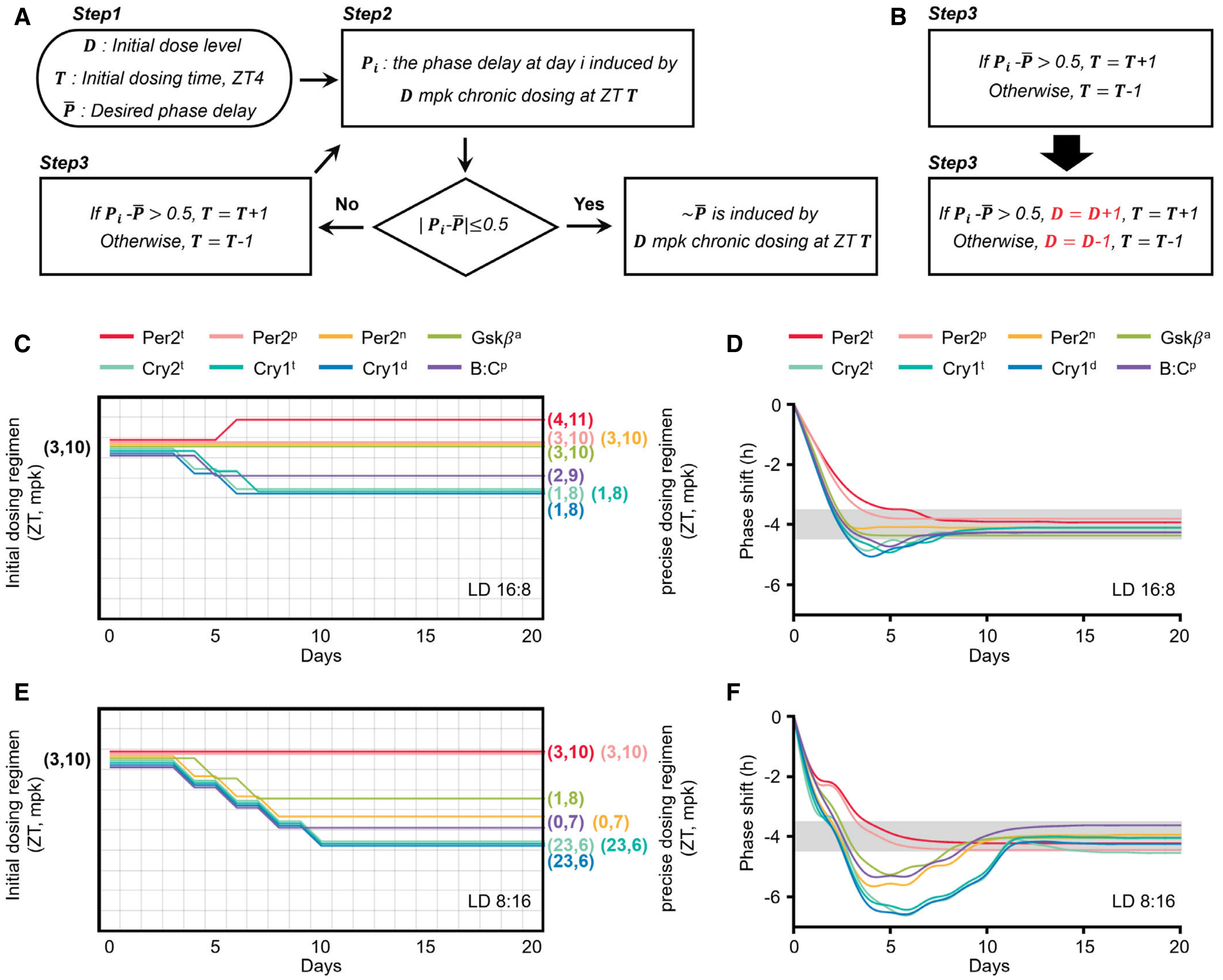

Figure EV6. Two types of adaptive chronotherapeutics.

A Detailed diagram describing an adaptive chronotherapeutics which identifies a precise dosing regimen by solely adjusting dosing time. *Step 1*: 10 mpk at ZT3 is recommended so that the following dosing time can be flexibly advanced or delayed depending on current drug effect with a low probability of leading to an accumulating or an alternating phase shift (Fig EV4B–D). *Step 2*: Single daily dosing is performed until a stable phase delay is observed (i.e., the phase delays at two consecutive days, $i-1$ and i, are similar, $|P_i - P_{i-1}| < 0.2$ h). However, if a larger phase delay than the desired one is achieved before the stable phase delay is obtained (i.e., $\bar{P} - P_i > 0.5$ h), the dosing is immediately stopped. *Step 3*: Depending on the drug effect observed in *Step 2*, the dosing regimen is modified (Fig 5A). If the induced phase delay is smaller than the desired phase delay (i.e., $P_i - \bar{P} > 0.5$ h), the dosing time is delayed by 1 h. Otherwise ($\bar{P} - P_i > 0.5$ h), the dosing time is advanced by 1 h. *Step 2* and *Step 3* are repeated until a constant stable phase delay similar to the desired one ($|P_i - \bar{P}| \leq 0.5$ h) is achieved.

B Modification of the adaptive chronotherapeutics (A) to adjust dose level as well as dosing time simultaneously: If the induced phase delay is smaller than the desired phase delay (i.e., $P_i - \bar{P} > 0.5$ h), the dose level is increased by 1 mpk and the dosing time is delayed by 1 h. Otherwise ($\bar{P} - P_i > 0.5$ h), the dose level is decreased by 1 mpk and the dosing time is advanced by 1 h.

C–F Due to the simultaneous change in dose level and dosing time (B), the precise dosing regimen can be more rapidly achieved with a lower dose level than the adaptive chronotherapeutics solely adjusting the dosing time (Fig 5B–E).

© 2019 The Authors
