# Supplementary material for: Systems approach reveals photosensitivity and PER2 level as determinants of clock‐modulator efficacy
Source: Mol Syst Biol. 2019 Jul 8;15(7):e8838. doi: 10.15252/msb.20198838 (PMC6613017; doi:10.15252/msb.20198838)
Supplement: Supplementary file 8 — Code EV1 [file MSB-15-e8838-s008.zip › MSB-19-8838_CodeEV1/readme_v5.docx]

1. The script *PF-670462_NHP_model.nb* includes the systems chronopharmacology model for diurnal non-human primates (NHPs) (Fig 2A), which simulates the effect of PF-670462, the CK1 inhibitor, and light on the circadian clock of NHPs.

Execution process:

A. Select the model, the dosing regimen of PF-670462 and lighting condition to be used for simulation by changing the values of parameters: *modeltype, select, days, dtt, dose* and *ldd* which determine the type of model (model N or W), the parameter set of gating and adaptation for light, dosing days, dosing time, dose level and day length, respectively.

C. Run cells, the basic units consisting MATHEMATICA code, before the “Simulation of chronic dosing under LD cycle” cell. This constructs the gating and adaption for light and incorporates them into the light module of the model.

D. Run the remaining cells, which simulate the phase delay of NHPs induced by PF-670462.

Description of each step is also included as explanatory notes in the code.

1. The script *ASPD_models.nb* includes the systems chronopharmacology model for advanced sleep phase disorder (ASPD) patients who have 4 h more advanced circadian phase than the healthy people.

Execution process:

A. Select genetic mutation, the dosing regimen of PF-670462 and lighting condition to be used for simulation by changing the values of parameters: *select, days, dtt, dose* and *ldd* which determine the type of mutation, dosing days, dosing time, dose level and day length, respectively.

B. Run cells before the “Simulation of chronic dosing under LD cycle” cell. This constructs the gating and adaption for light and incorporates them into the light module of the model.

C. Run the remaining cells, which simulate the phase delay induced by PF-670462.

Description of each step is also included as explanatory notes in the code.

1. The script *parameter_estimation_simulated_annealing.nb* was used to estimate the parameter set describing the shape of gating and adaption for light (Fig EV1F and H) with which the model accurately simulates the phase delay of NHPs induced by the 3-day LD dosing and the magnitude of human PRC to a 6.7 h light pulse (Fig EV2A), via simulated annealing. Through the estimation, the 911 parameter sets were identified, which were stored in the *data* folder. The simulated LD dosing effect and the simulated phase response curves (PRCs) with the 911 parameter sets to a 12 h light pulse and 6.7 h light pulse, 3-cycle 5 h light pulses were also stored in the *data* folder.
2. The script *parameter_estimation_post_filtering.nb* was used to identify the parameter sets among the 911 estimated parameter sets with which the model accurately simulates the type 1 PRC to a 12 h light pulse and human PRC to a 6.7 h light pulse and to 3-cycle 5 h light pulses (Fig EV2B-E). Through the post filtering, 10 parameter sets were finally identified (Fig EV2F).

Reference: Kim DW, Chang C, Chen X, Doran A, Gaudreault F, Wager T, DeMarco GJ, Kim JK, Systems approach reveals photosensitivity and PER2 level as determinants of clock-modulator efficacy
